# Scalable agroinfiltration-based production of SARS-CoV-2 antigens for use in diagnostic assays and subunit vaccines

**DOI:** 10.1371/journal.pone.0277668

**Published:** 2022-12-14

**Authors:** Jordan Demone, Mariam Maltseva, Maryam Nourimand, Mina Nasr-Sharif, Yannick Galipeau, Emilio I. Alarcon, Marc-André Langlois, Allyson M. MacLean

**Affiliations:** 1 Department of Biology, University of Ottawa, Ottawa, Ontario, Canada; 2 Department of Biochemistry, Microbiology and Immunology, University of Ottawa, Ottawa, Ontario, Canada; 3 BEaTS Research, Division of Cardiac Surgery, University of Ottawa Heart Institute, Ottawa, Ontario, Canada; 4 University of Ottawa Centre for Infection, Immunity and Inflammation (CI3), Ottawa, Ontario, Canada; Waseda University: Waseda Daigaku, JAPAN

## Abstract

Agroinfiltration is a method used in biopharming to support plant-based biosynthesis of therapeutic proteins such as antibodies and viral antigens involved in vaccines. Major advantages of generating proteins in plants is the low cost, massive scalability and the rapid yield of the technology. Herein, we report the agroinfiltration-based production of glycosylated SARS-CoV-2 Spike receptor-binding domain (RBD) protein. We show that it exhibits high-affinity binding to the SARS-CoV-2 receptor angiotensin-converting enzyme 2 (ACE2) and displays folding similar to antigen produced in mammalian expression systems. Moreover, our plant-expressed RBD was readily detected by IgM, IgA, and IgG antibodies from the serum of SARS-CoV-2 infected and vaccinated individuals. We further demonstrate that binding of plant-expressed RBD to ACE2 is efficiently neutralized by these antibodies. Collectively, these findings demonstrate that recombinant RBD produced via agroinfiltration exhibits suitable biochemical and antigenic features for use in serological and neutralization assays, and in subunit vaccine platforms.

## Introduction

Agroinfiltration is a well-established method used frequently in plant biology in which a strain of the Gram-negative alpha-Proteobacterial species *Agrobacterium tumefaciens* is ‘injected’ with a needleless syringe into plant leaves. This bacterial plant pathogen then employs a specialized secretion system to genetically transform plant host nuclei by transferring genes of interest into recipient host cells with a high degree of efficiency. The transient transformation of *Nicotiana benthamiana* via agroinfiltration is one of the most rapid methods to efficiently express recombinant proteins in any eukaryotic system [1]. Unlike traditional stably transformed transgenic plants that may require many months to generate, we can transiently transform the leaves of *N*. *benthamiana* to (co-)express one or multiple proteins simultaneously, observing high levels of protein expression in 3–4 days. This system is highly responsive and flexible. It is well-suited for supporting the rapid development of viral antigen-based diagnostic tests, such as serological assays to detect antibodies in blood, and vaccines against diseases such as COVID-19 in real time where the occurrence of viral variants represent an ever-evolving target.

In this work, we describe the development of a rapid and flexible agroinfiltration-based platform for the production of recombinant SARS-CoV-2 Spike Receptor-Binding Domain (RBD) in the plant *Nicotiana benthamiana*. This platform is simple, massively scalable, cost-effective and easily adaptable to reflect rapid changes in circulating viral sequences. The RBD of the viral spike is the region primarily involved in binding to the cell surface receptor of the virus, the angiotensin converting enzyme 2 (ACE2) receptor [2]. Most neutralizing antibodies against SARS-CoV-2 are directed to RBD [3–5]. A number of studies have already demonstrated the feasibility of plant-based RBD antigen development: histidine-tagged RBD has been expressed at levels as high as 8 ug/g leaf biomass [6], and similar RBD antigens exhibit effective neutralizing responses in mice [7] and non-human primates [8].

**Fig 1:**
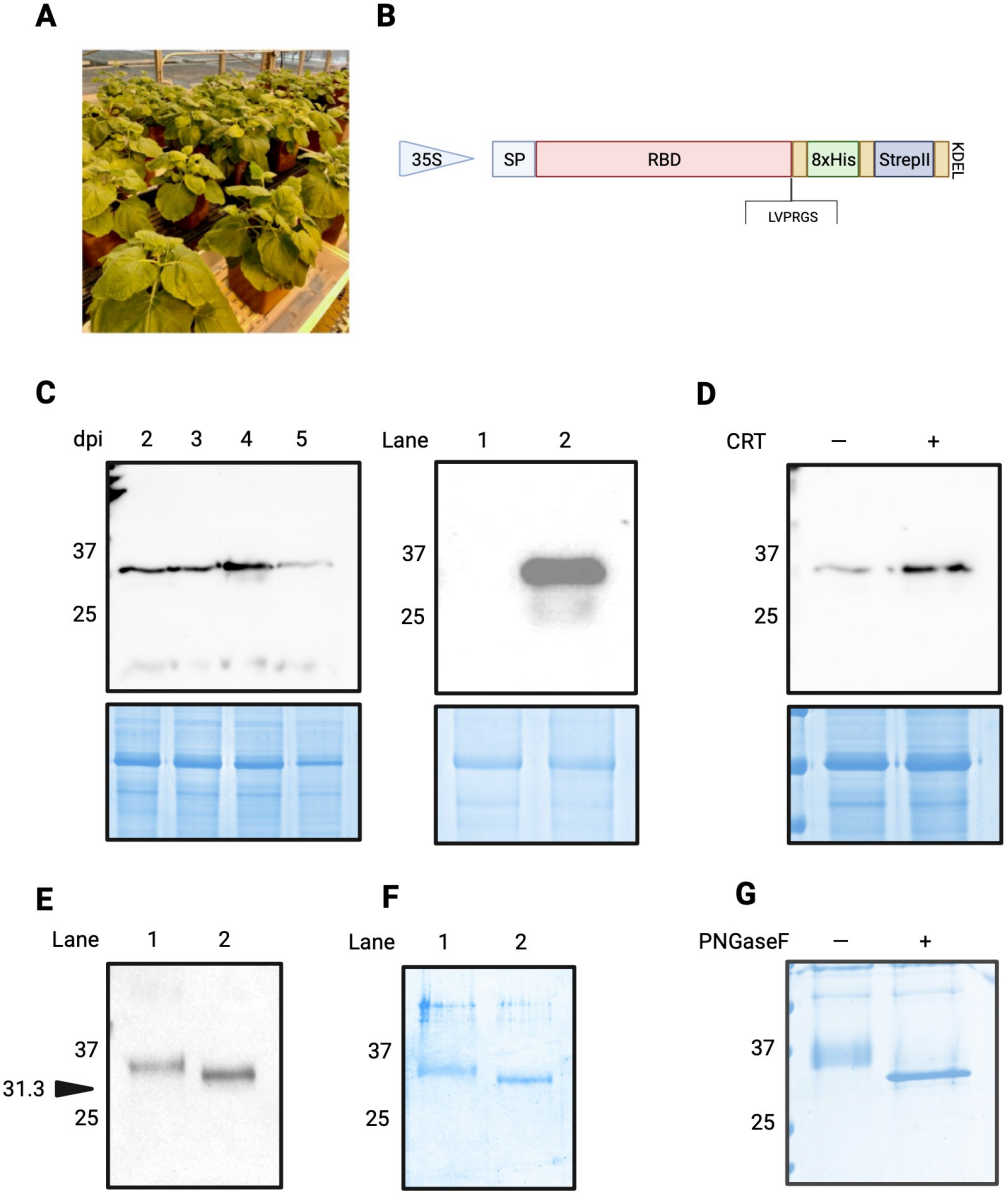
Expression and purification of SARS-CoV-2 RBD in *Nicotiana benthamiana*. (A) Agro-infiltrated *Nicotiana benthamiana* growing in greenhouse. (B) Schematic representation of genetic construct used to express SARS-CoV-2 RBD *in planta*. The SARS-CoV-2 sequence was expressed as a recombinant protein with a dual 8xHis and Twin-Strep II tag, interspersed with Gly-Ser linkers (gold boxes). An ER-retention KDEL sequence was positioned at the C-terminus, and a Thrombin cleavage site (LVPRGS) was included for tag removal. (C) Left panel: Anti-His IB of samples obtained from *N*. *benthamiana* 2 to 5 days post-infiltration (dpi) with RBD construct in B. Loading control at 5 dpi reproducibly demonstrates reduced abundance of protein due to initiation of tissue necrosis at this time. Right panel: NT control (lane 1) compared to RBD expressed in *N*. *benthamiana* with calreticulin (lane 2). Anti-his IB. (D) Co-infiltration of human calreticulin (CRT) increases expression levels of RBD in *N*. *benthamiana*. Samples collected 4dpi. Anti-his IB. (E) Anti-S1 IB of purified RBD expressed in *N*. *benthamiana* (lane 1) and control RBD expressed in mammalian 293F cells (lane 2). Arrow indicates expected migration of a protein corresponding to 31.3 kDa. (F) CBB-stained SDS-PAGE of purified RBD expressed in *N*. *benthamiana* (lane 1) and control RBD expressed in mammalian 293F cells (lane 2). (G) CBB-stained SDS-PAGE of plant-derived RBD treated with (+) and without (-) the amidase Peptide-N-Glycosidase F (PNGaseF), an enzyme that cleaves *N*-linked glycan chains. SP, signal peptide. IB, immunoblot. CBB, Coomassie Brilliant Blue. NT, non-transformed.

The system we describe here utilizes *Agrobacterium tumefaciens*-mediated transient transformation of *N*. *benthamiana* (Fig 1), to enable the biosynthesis of high-quality SARS-CoV-2 RBD antigen *in planta*. This accessible and cost-effective process requires approximately only six weeks, encompassing seed germination to delivery of the tandem-purified antigen. We demonstrate in this study that plant expressed RBD displays similar biochemical, structural and antigenic properties as RBD produced in classical mammalian cell expression systems, thereby indicating its suitability for use in diagnostic tests.

Aside from diagnostics assays, SARS-CoV-2 antigens can also be utilized in adjuvanted subunit vaccines [9–11], as well as unadjuvanted vaccines when used as a booster shot [12]. While most SARS-CoV-2 vaccines focus on intramuscular delivery systems, development of a subunit nasal spray vaccine offers a complementary method to increase mucosal immunity to SARS-CoV-2 variants and provide vaccine-hesitant and needle-phobic individuals with alternative immunization options [13]. Protein subunit vaccine platforms have been extensively characterized and have an established safety and efficacy profile against multiple pathogens such as Shingles, Hepatitis B and Meningitis B [14]. The effectiveness of plant-expressed proteins in vaccines has been demonstrated in animal models where they induced potent mucosal immune responses and protection following intranasal immunization [15, 16]. Vaccines produced in plants offer several advantages over traditional manufacturing processes, including significantly lower production costs, increased scalability, and a negligible risk of contamination with human pathogens [17]. These advantages are critical parameters towards facilitating equitable vaccine access to the world’s population.

## Materials and methods

### Plant materials and growth conditions

Wild-type *Nicotiana benthamiana* plants were grown in a greenhouse equipped with 400-Watt high pressure sodium (HPS) light bulbs set at a 20h light/4h dark photoperiod at 25°C for five weeks prior to infiltration. Plants were fertilized once per week using Miracle-Gro (24-8-16) at a concentration of 1.1 g/L. Seeds were germinated on a weekly basis and 56 two-week-old seedlings were transplanted to individual pots once a week.

### Gene synthesis

The RBD sequence was obtained from the Wuhan strain (NC_045512) Spike sequence spanning amino acids 319 to 540. The sequence designed for expression in *N*. *benthamiana* had a C-terminal thrombin cleavage site, 8xhis tag, a Twin-Strep-tag^®^, and a KDEL sequence for ER-retention. An endoplasmic reticulum targeting signal peptide derived from *Nicotiana tabacum* PR-1a signal peptide (amino acids 1 to 31) was added to the N-terminal side of the RBD sequence. The construct was codon-optimized for *N*. *benthamiana* expression and was synthesized by GenScript into the pHREAC vector [18] via BsaI sites. Lectin-binding human chaperone calreticulin (NP_004334.1) was codon-optimized for *N*. *benthamiana* and synthesized in the pHRE vector [18].

### Transient expression in *Nicotiana benthamiana*

Prior to infiltration, pHREAC-RBD and pHRE-calreticulin were freshly transformed into *Agrobacterium tumefaciens* strains AGL1 and Gv2260, respectively, via electroporation. A single colony was used to inoculate each 5 mL starter culture, and each culture was sub-cultured once in two litres of LB broth containing 50 mg/L ampicillin and 50 mg/L kanamycin at 28°C. The final cultures were grown to an optical density at 600nm (OD600) of 0.6 and were centrifuged and resuspended in MMA buffer (10 mM MES, 10 mM MgCl_2_, 200 μM acetosyringone, pH 5.6). Unless otherwise indicated, *Agrobacterium* containing the pHREAC-RBD and pHRE-calreticulin constructs were combined and incubated at room temperature for one hour prior to infiltration, to a final OD600 of 0.25 (pHREAC-RBD) and 0.1 (pHRE-CRT). Small-scale infiltration was performed by using 1 mL needleless syringes to inject *Agrobacterium* into the abaxial side of the leaves. Large-scale infiltration was performed via vacuum infiltration using three litres of infiltration solution. Leaves were gently scored, and individual plants were inverted and placed into the vacuum chamber for 2–3 minutes at a pressure of 5 bar. The vacuum was repeatedly applied until the plant was fully infiltrated. Infiltrated leaves were marked and plants were returned to the greenhouse until collection. Twenty-five plants were typically infiltrated per large-scale infiltration, equating to approximately 200 grams of fresh leaf biomass.

### Total soluble protein extraction

Leaves co-infiltrated with pHREAC-RBD and pHRE-calreticulin were collected 4–5 dpi (days post-infiltration). Leaf spines were removed, and tissue was homogenized in liquid nitrogen with mortar and pestle. Protein extracts were prepared under native conditions. For each FPLC run: 60 grams of ground tissue were resuspended in 270 mL of ice-cold buffer (PBS (20 mM NaH_2_PO_4_, 280 mM NaCl, 6 mM KCl), 10 mM imidazole, pH 7.4) and vortexed for five minutes. Ten microlitres of Lysonase Bioprocessing Reagent (Sigma Aldrich Canada Ltd; Etobicoke, Ontario) was added and the lysate was incubated at 4°C for 15 minutes while gently shaking. Lysate were vortexed again and sonicated three times for 1 minute using a Kontes micro ultrasonic cell disruptor set at 70 output. Lysate was centrifuged for 45 minutes at 20,442 x *g* at 4°C to pellet cell debris. Supernatants were filtered using several layers of cheesecloth and subsequently filtered using Corning one liter 0.22 μM polyethersulfone (PES) sterilizing low-binding filters (Corning, Incorporated; Corning, NY, USA).

### Purification of RBD using HisPur^™^ Ni-NTA resin

Clarified total protein extract was passed through a HisPur Ni-NTA resin column (5 mL bed volume; Cytiva, Marlborough, US), pre-equilibrated with five volumes of imidazole binding buffer (PBS, 10 mM imidazole buffer, pH 7.4) using a fast protein liquid chromatography (FPLC) system (AKTA Pure; GE Healthcare systems). The column was washed with twenty bed volumes of binding buffer (PBS, 10 mM imidazole, pH 7.4) at a flow rate of 5 mL/minute. Protein was eluted using five bed volumes of elution buffer (PBS, 500 mM imidazole, pH 7.4) at a flow rate of 2 mL/minute. Samples were collected as 2 mL fractions. In initial experiments, aliquots of each fraction were visualized via immunoblotting (anti-His antibody; Cat: SAB2702218, Sigma Aldrich Canada, Oakville, Canada) to identify fractions enriched for RBD, which we found to consistently correspond to a visible peak eluting from the column in fractions 25 to 35.

### Purification of RBD using Strep-Tactin resin

Fractions corresponding to the protein peak from the HisPur Ni-NTA resin were pooled and protein was further purified using a Strep-Tactin resin column (5 mL bed volume; Cytiva, Marlborough, US) using the AKTA Pure system. The Strep-Tactin column was pre-equilibrated with five bed volumes of binding buffer (PBS, pH 7.4) prior to sample loading, and the column was then washed with 10 bed volumes of binding buffer (PBS, pH 7.4). Protein was eluted using six bed volumes of StrepTrap elution buffer (PBS, 5 mM *d*-Desthiobiotin, pH 7.4) at a flow rate of 2 mL/minute. Samples were collected as 0.5 mL fractions. In initial experiments, aliquots of each fraction were immunoblotted (probed with anti-His antibody) and protein was also visualized via SDS-PAGE gels stained with Coomassie blue staining solution. We reproducibly detected RBD within fractions 22 to 33, which corresponded to the visible peak eluting from the column. Peak samples were collected, pooled, and concentrated to 500 μL using a Vivaspin 20 column (GE Healthcare; Mississauga, Canada). Plant and mammalian RBD were run on an SDS-PAGE gel and Coomassie-stained for analysis. Both proteins were also immunoblotted with anti-S1 (1: 2000) antibody (Cat: PA5-81795, Invitrogen) and a goat anti-rabbit secondary antibody (1: 10 000).

### Further processing of RBD protein

Thrombin cleavage was performed to remove the tag from purified RBD protein. Ten microlitres of thrombin (1U/μL) were added to the 500 μL sample of purified RBD protein and incubated at 22°C for 20 hours. To remove the cleaved 3 kDa tag from the final product, the reaction was passed through a Strep-Tactin resin column (5 mL bed volume) as described above. Flowthrough fractions corresponding to the peak were collected and concentrated to 500 μL using a Vivaspin 20 column (GE Healthcare, Mississauga, Canada). Protein concentration was quantified using the Bio-Rad Protein Assay and used BSA for standard curve preparation. Protein purity was analyzed using SDS-PAGE and protein was visualized using Coomassie blue staining solution.

### PNGase F assay

PNGase F, an amidase, is an enzyme used to cleave *N*-linked glycans from glycoproteins and was used to assess the glycosylation status of plant-expressed RBD. Briefly, 20 μg of partially purified plant RBD protein (following elution from the StrepTrap column) was incubated with denaturation buffer at 100°C for 10 minutes. The sample was chilled to halt the denaturation process and GlycoBuffer 2 (New England Biolabs), NP-40, and PNGase F were added to the sample. The sample was incubated at 37°C for one hour and analyzed on an SDS-PAGE gel. Bands were visualized using a Coomassie blue staining solution.

### Circular dichroism (CD) spectra and secondary structure calculations

CD spectra were recorded in a J-810 CD spectrophotometer from Jasco analytical instruments equipped with a temperature-controlled Peltier system at 37±0.2°C. Spectra were measured in 0.2 mm path length cuvettes (50 μL of sample) and recorded using a scan rate of 100 nm/min, in high sensitivity mode between 200–250 nm (1.0 nm intervals). Total number of accumulations per spectra was set as five. The equipment is routinely calibrated with camphorsulfonic acid. A mammalian RBD standard was used as a reference protein for the CD analysis and secondary structure calculation (Cat: Pro1151-3, National Research Council, Ottawa, Canada). Secondary structural analyses were carried out using the BeStSel analysis server [19].

### Indirect ELISA to assess ACE2 binding (Kd measurement)

To evaluate protein binding, an indirect ELISA was carried out. Plant or mammalian produced SARS-CoV-2 RBD protein were diluted in sterile 1X PBS (Multicell #311-010-CL) to 4 μg/mL and coated onto a 384-well Immuno plates (Thermofisher, #460372) (12.5 μL/well) overnight at 4°C. Plates were washed three times with 100 μL of PBS-T (PBS, 1% Tween-20) using a BIOTEK plate washer (model ELX405) and blocked for one hour with blocking buffer (PBS-T + 3% non-fat milk powder, w/v) while shaking (700rpm) at room temperature. Biotinylated ACE2 as produced in Abe et al., was diluted in dilution buffer (PBS-T + 1% non-fat milk powder, w/v) to 258 ng/μL and subsequently 1:2 serially diluted [20]. Plates were washed thrice with PBS-T, followed by addition of 20 μL per well of titrated soluble ACE2 and incubated for one hour while shaking at room temperature. Plates were again washed thrice with PBS-T followed by the addition of 20 μL per well of Streptavidin-Peroxidase polymer (Sigma #S2438) diluted in dilution buffer to 1.25 ng/μL. After a one hour incubation with shaking, plates were washed thrice with PBS-T and 20 μL of freshly prepared SuperSignal ELISA Pico Chemiluminescent Substrate (Thermo Scientific, #37069) (mixed 1:1 ratio and diluted in equal volume with dH_2_O, (V:V)) was added to each well. Following 5 mins of incubation on a shaker, the luminescence signal (relative light units; RLU) was measured with BIO-TEK Synergy Neo2 plate reader at 20 ms/well at a read height of 1.0 mm. Wells filled with dilution buffer in place of ACE2 accounted for background luminescence and were subtracted from the titrated ACE2 values. Dissociation constant (K_D_) was determined using 4-parameter curve fitting with GraphPad Prism 9.1.2 software.

### Indirect ELISA to evaluate anti-SARS-CoV-2 immunoreactivity in serum samples (serology)

Plant or mammalian produced SARS-CoV-2 RBD protein were diluted in sterile 1X PBS to 2 μg/mL and coated onto a 96-well plates (VWR #62402–959) (50 μL/well) overnight at 4°C. Plates were washed three times with 200 μL of PBS-T and blocked for one hour with a blocking buffer (PBS-T + 3% non-fat milk powder, w/v) on a shaker at room temperature. Serum samples were diluted 1:50 in dilution buffer (PBS-T + 1% non-fat milk powder, w/v). In conjunction, titration curves of conformation-dependent monoclonal IgM (Absolute Antibody, Ab01680-15.0), IgA (Absolute Antibody, Ab01680-16.0), and IgG (Absolute Antibody, Ab01680-10.0) CR3022 antibodies were used as reference material to assess protein folding. CR3022 antibodies were diluted 1:2000, followed by 1:2 serial dilution to establish a calibration curve. After blocking, plates were washed thrice with PBS-T, and followed by addition of 100 μL of the respective diluted serum samples and CR3022 antibodies. The plates were incubated for two hours on a shaker at room temperature, washed thrice with PBS-T followed by the addition of 50 μL of the respective secondary-HRP antibody at specified dilutions (1:4000 secondary anti-human IgG-HRP (NRC anti-hIgG#5-HRP fusion), anti- human 1:8000 IgA-HRP (Jackson ImmunoResearch, 109-035-011) or 1:9600 anti- human IgM-HRP (Jackson ImmunoResearch, 109-035-129)). Plates were incubated for one hour on a shaker, washed thrice with PBS-T followed by the addition with 100 μL of the diluted SuperSignal ELISA Pico Chemiluminescent Substrate. Luminescence intensity was measured with BIO-TEK Synergy Neo2 plate reader for 20ms/well at a read height of 1.0 mm. Wells filled with dilution buffer in place of serum accounted for background luminescence and were subtracted from the patient serum values.

### Surrogate neutralization ELISA (snELISA) assay to evaluate neutralization activity in serum samples

The described methodology was adapted from the surrogate neutralization ELISA assay as shown in Abe *et al*. (2020) for the evaluation of the relative inhibition of neutralizing antibodies to RBD protein from binding to soluble ACE2 [20, 21]. Briefly, plant or mammalian produced SARS-CoV-2 RBD protein were diluted in sterile 1X PBS to 8 μg/mL and coated onto a 384-well Immuno plates (12.5 μL/well) overnight at 4°C. Plates were washed three times with PBS-T and blocked for one hour while shaking. Serum samples were 1:5 serially diluted in a dilution buffer, applied to wells (20 μL/well) and incubated for 2 hours while shaking at room temperature. Plates were washed thrice, followed by the addition of biotinylated ACE2 diluted to 0.35 ng/μL in a dilution buffer (20 μL/well). Plates were washed thrice with PBS-T followed by the addition of 20 μL per well of Streptavidin-Peroxidase polymer diluted in dilution buffer to 1.25 ng/μL. Following one hour incubation, plates were washed thrice with PBS-T and freshly prepared SuperSignal ELISA Pico Chemiluminescent Substrate was applied (20 μL/well). Following 5 mins of incubation while shaking, luminescence intensity was measured with BIO-TEK Synergy Neo2 plate reader for 20ms/well at a read height of 1.0 mm. Control wells were filled with a dilution buffer in place of serum followed by the addition of ACE2 to assess maximum binding signal. Relative percent inhibition was calculated as follows:

$$\%\;Inhibition=\left(1-\frac{average\;mean\;of\;serum\;sample}{average\;mean\;of\;maxium\;signal}\right)\times 100$$

Serum dilution resulting in a 50% inhibition (half-maximal inhibitory dilution (ID_50_)) of RBD protein from binding ACE2 receptor was determined using 4-parameter fitting with GraphPad Prism 9.1.2 software.

### RBD production in mammalian cells

For comparison, a mammalian RBD was produced in HEK 293F cells and purified. Briefly, a plasmid generously provided by Dr Florian Krammer (Mount Sinai, NYC) encoding the Whuhan-Hu-1 RBD (MN908947) sequence coding for the amino acid 319–541 and fused with the N-terminal SARS-CoV-2 spike secretory signal and a C-terminal hexa-histidine tag was transfected into 293F cells cultivated in Freestyle 293 expression media (Thermo Fisher, #12338018) at 37°C, 7% CO_2_, while shaking (125rpm). A total of 600 million cells resuspended in 200 mL were transfected with 200 μg of plasmid using ExpiFectamine (Thermo Fisher, 14525). Three days post-transfection, cell supernatant was harvested by centrifugation (4000xg for 20 mins at 4°C) and filtered through a low binding 0.22 μm stericup vacuum filter (Millipore Sigma, S2GPU10RE). The filtered supernatant was incubated for 2 hours at room temperature with 6ml of Ni-NTA resin (Qiagen, 30210). The column containing the mix of supernatant and resin was washed four times with a washing buffer containing 20mM imidazole, 300mM NaCl and 57.5mM of NaH_2_PO_4_·H_2_O. The RBD protein was then eluted with three column volumes of the elution buffer containing 234mM of imidazole, 300mM NaCl and 57.5mM of NaH_2_PO_4_·H_2_O. The eluted solution was concentrated and the buffer was replaced with PBS using a 10kDa Amicon filter (Millipore Sigma, UFC901008). RBD protein integrity was verified by SDS-PAGE, aliquoted to minimize freeze-thaw cycles and stored at -80°C. RBD used for CD analysis was produced by the National Research Council of Canada and was a gift from Yves Durocher. RBD production details are published elsewhere [21].

### Patient samples and collection

Use of human samples for this study was approved by the University of Ottawa Ethics Review Board: Certificates H-04-20-5727, H-04-21-6643 and H-07-20-6009. All participants gave informed written consent to participate in the study. The negative sample was taken from an individual with no history of SARS-CoV-2 infection and tested with PCR. Pooled negative samples were obtained from individuals negative for SARS-CoV-2 as tested by PCR and serology assay. Serum samples from convalescent or vaccinated patients enrolled in surveillance studies from different research studies post-2019. Samples were collected using standard phlebotomy procedures. Samples were de-identified and held at 4°C for short term handling and testing at University of Ottawa CL2+ biocontainment facility. All research was performed in accordance with current guidelines and regulations.

## Results

A codon-optimized RBD sequence corresponding to the Wuhan-Hu-1 isolate of SARS-CoV-2 was cloned into the pHREAC vector backbone [18] a plant-specific expression platform that has been demonstrated to achieve among the highest levels of *in vivo* protein expression reported in *N*. *benthamiana* (Fig 1B). The RBD sequence was flanked on the N-terminal side by an endoplasmic reticulum targeting signal peptide derived from *Nicotiana tabacum* PR-1a signal peptide (amino acids 1–31), and on the C-terminal side by a tandem affinity tag consisting of an 8xHis (OctoHis) tag and a Twin-Strep-tag (Fig 1B). The dual affinity tags were separated by a glycine-serine linker, flanked by a thrombin cleavage site (LVPRGS) that was positioned directly upstream of the affinity tags, and an ER retention signal (KDEL) located downstream and adjacent to two tandem stop codons. For an initial assessment of expression, samples were flash frozen in liquid nitrogen, ground, and resuspended in SDS-PAGE loading buffer. These crude samples were next analyzed using immunoblot analysis (Fig 1C & 1D) prior to purification.

Initial screening of the sequence validated expression vector demonstrated that the greatest expression was achieved using *Agrobacterium tumefaciens* strain AGL1 when infiltrated into five-to-six-week-old *N*. *benthamiana* leaves that were collected four days post-infiltration (dpi) (Fig 1C, left panel), with lower levels of protein expression also observed at 2-, 3-, and 5 dpi. At 3dpi leaves began to develop chlorosis and subsequently reduced photosynthesis, becoming increasingly necrotic 5dpi onward. We did not observe a signal in NT (non-transformed) plants when crude extracts were immunoblotted with anti-His (Fig 1C, right panel). Margolin et al. (2019) had previously demonstrated much enhanced expression of a trimeric Spike mimetic protein in *N*. *benthamiana* when co-expressed in the presence of human calreticulin [22] thus we assessed expression of RBD in the presence and absence of this chaperone. Co-infiltrating with calreticulin (CRT) produced a noticeable increase in RBD expression levels (Fig 1D) and we thus opted to co-express calreticulin with RBD in subsequent infiltrations, with the assumption that the human chaperone may promote proper RBD folding. All RBD samples revealed a band that migrated slightly above the expected molecular weight of 31.3 kDa. Plant-expressed RBD migrates slightly above the mammalian-expressed RBD control (Fig 1E & 1F), possibly due to differences in glycosylation profiles. Immunoblot analysis using an antibody raised against the SARS-CoV-2 S1 region of the Spike protein confirmed the identity of both plant and mammalian RBD. The S1 antibody also recognized a band at ~75 kDa for the plant RBD sample only, suggesting homodimer formation.

To scale up purification, we agroinfiltrated production batches of 25–30 five-to-six-week old plants (Fig 1A) equivalent to approximately 200 grams fresh biomass) under a vacuum. As described in the methods, protein was partially purified by passing lysate through a Ni-NTA column, corresponding to purification of the 8xHis moiety of the tag. Fractions corresponding to the peak detected at 280 nm were visualized using immunoblot analysis to confirm presence and integrity of protein and were then loaded onto a StrepTrap column. Peak fractions were visualized via immunoblot, pooled, concentrated, and digested overnight with thrombin to remove the 8xHis-Twin-Strep-tag. The digested sample was passed through the StrepTrap column a second time to remove the cleaved tag, and aliquots of the purified protein were analyzed on a Coomassie gel (Fig 1F). A band on the Coomassie gel was visualized migrating at approximately 35 kDa, corresponding to the band recognized on the RBD immunoblot by an anti-S1 antibody (Fig 1E). We next assessed the glycosylation status of the plant and mammalian RBD antigens: the Spike protein of SARS-CoV-2 has 22 *N*-linked glycosylation sites, two of which are located within RBD [23]. Peptide-*N*-glycosidase F (PNGase F) digestion of the plant-expressed RBD protein confirmed that it was glycosylated by a shift in band size (Fig 1G). We estimate the purification yield of RBD at up to 10 ug per gram of fresh leaf mass, in line with other *in planta* production systems that also utilize a histidine-tag [6, 24].

**Fig 2:**
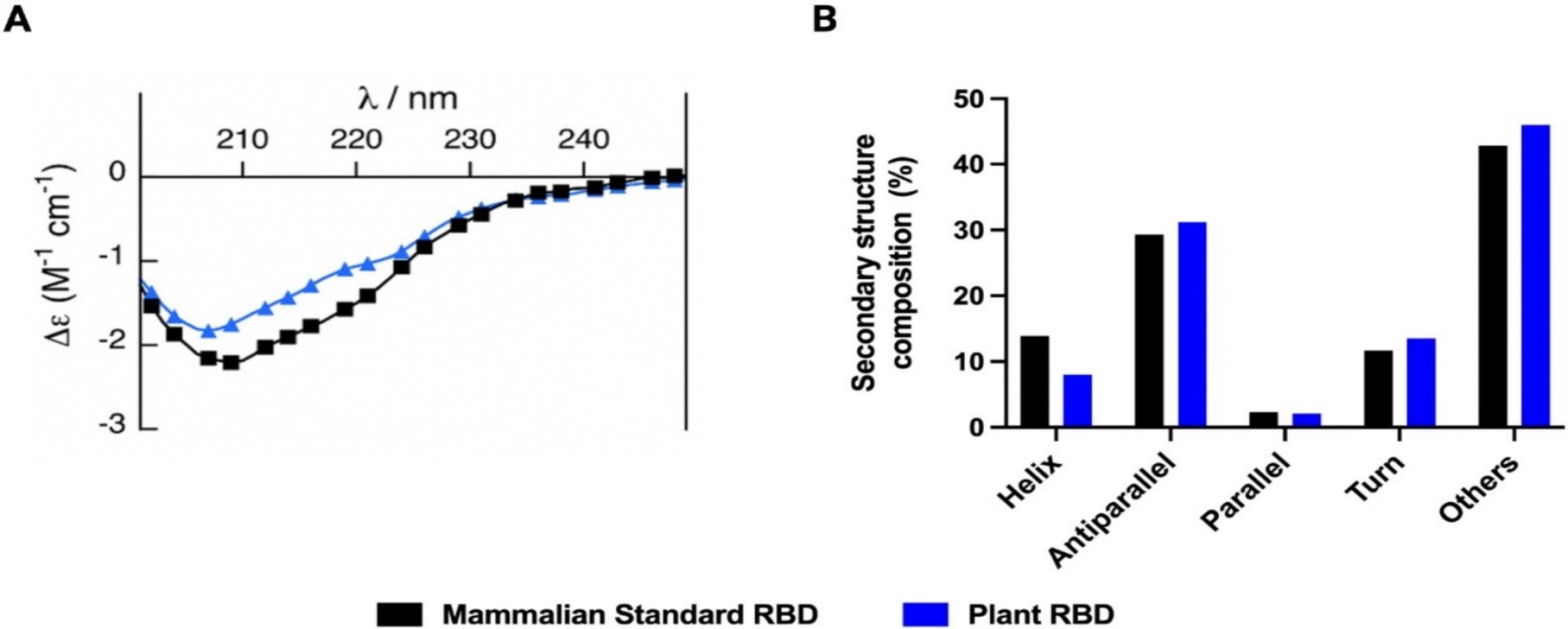
Biochemical evaluation of plant-expressed RBD by circular dichroism (CD). A) Spectral profiles of mammalian standard (black) and plant (blue) expressed RBD in PBS buffer pH 7.4 at 37°C (200–250 nm). Data expressed in molar circular dichroism (Δε) calculated from averaging 5 independent spectra of each sample. B) Secondary structure content in percentage calculated for mammalian and plant-derived RBD in PBS buffer pH 7.4 at 37°C (200–250 nm). Content was calculated using BeStSel analysis server by direct analysis of the raw data (mDeg) from the CD system. Molecular weights for both proteins were considered as 35 kDa.

We next performed a comprehensive functional assessment of the RBD produced in *N*. *benthamiana* in comparison to RBD produced in mammalian cells. Appropriate folding of the RBD antigen is critical for its interaction with the human ACE2 receptor and for the development of antibodies that will target and neutralize native virus and viral variants. We characterized the biochemical properties of the plant-expressed RBD by circular dichroism spectra (CD). More specifically we compared the folding of plant RBD to mammalian expressed RBD standard. We show that the spectral profile of plant-expressed RBD is largely similar to the mammalian expressed RBD, both displaying a single minimum at ~206 nm, consistent with previously reported profiles [25, 26] (Fig 2A). This is further reflected in the analysis of the secondary structure composition by BeStSel analysis server, which shows an overall similar distribution with small differences in α-helical content estimation, which could be due to different post-translational modifications between plant and mammalian cells (Fig 2B).

**Fig 3:**
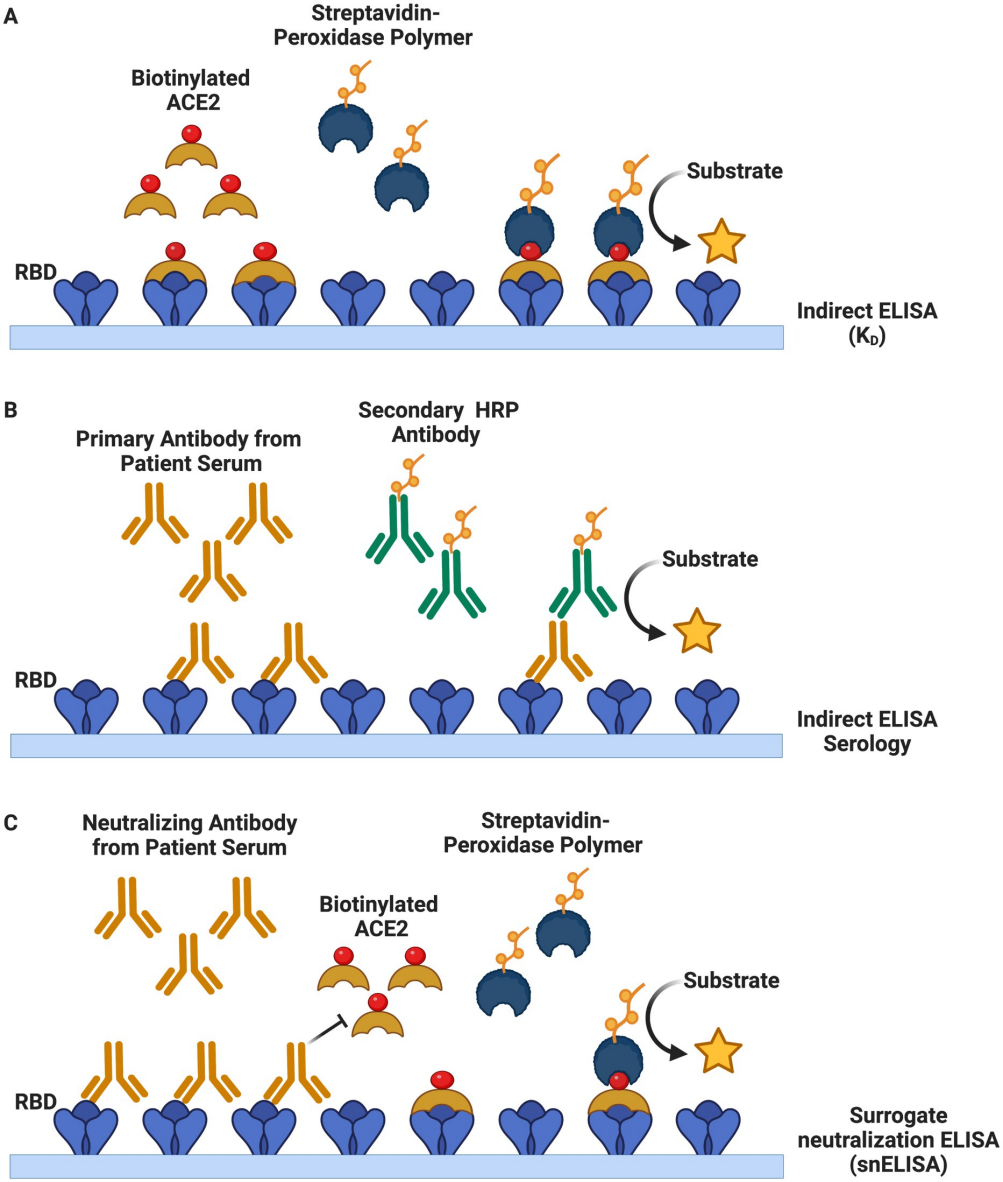
Schematic representations of the different types of ELISA used to characterize plant-expressed RBD. (A) Indirect ELISA (Kd) set up for the evaluation of binding kinetics of soluble human ACE2 to immobilized RBD. (B) Indirect ELISA (serology) set up for the evaluation of binding and recognition of commercial monoclonal and serum IgG, IgM and IgA antibodies to immobilized RBD. (C) Surrogate neutralization ELISA (snELISA) set up to evaluate relative inhibition of anti-SARS-CoV-2 neutralizing antibodies in blocking immobilized RBD from binding soluble ACE2.

**Fig 4:**
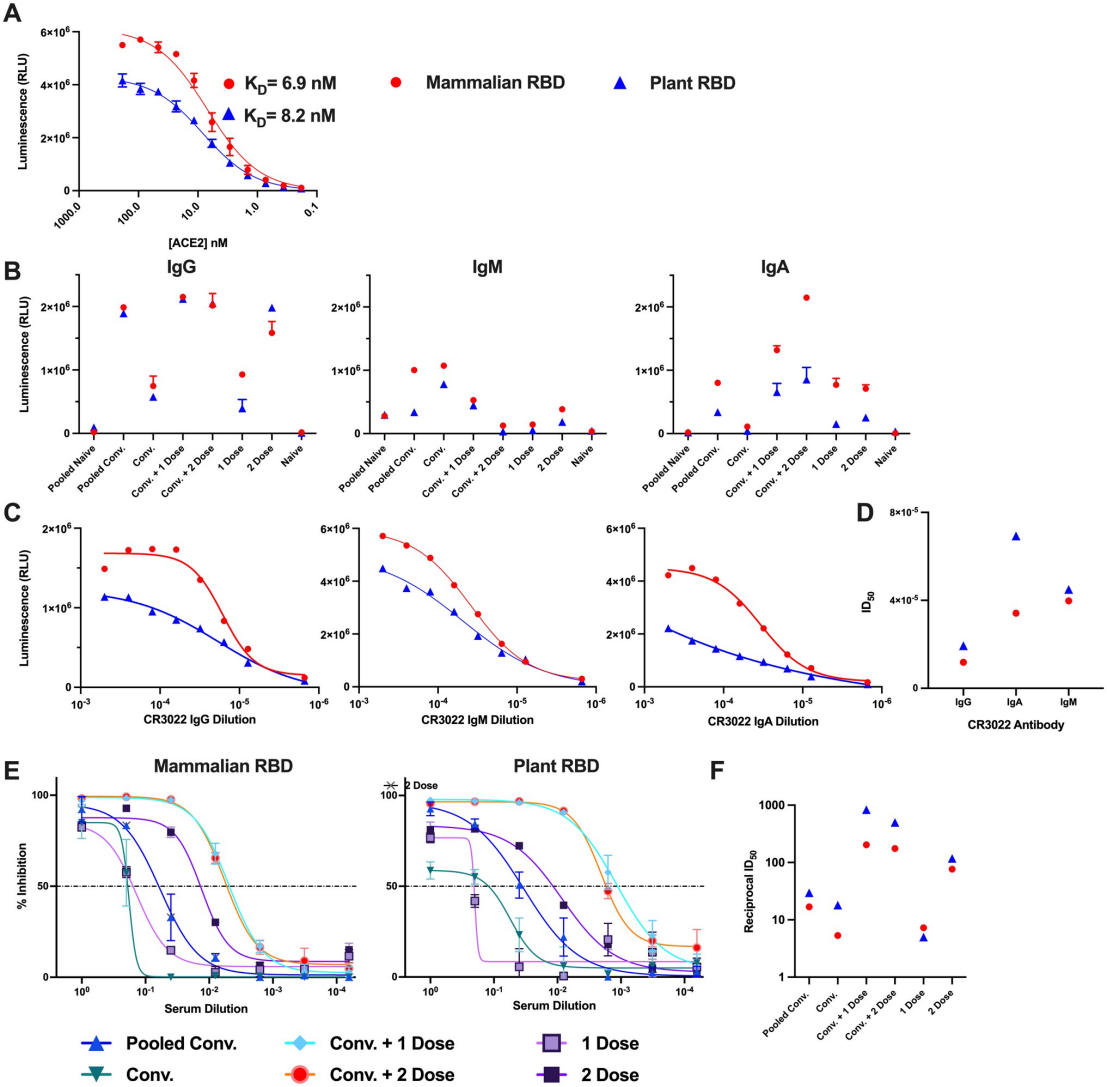
Evaluation of plant-expressed RBD protein functionality, folding and binding kinetics. A comprehensive assessment of protein function of the RBD produced in *N*. *benthamiana* as it pertains to protein folding and binding to ACE2 receptor, recognition and neutralization by antibodies in sera from SARS-CoV-2 exposed individuals. (A) Indirect ELISA demonstrating binding kinetics of soluble human ACE2 to immobilized mammalian RBD (red circle) and plant RBD (blue triangle). (B) Binding and recognition of immobilized mammalian and plant produced RBD by IgG, IgM and IgA polyclonal antibodies in sera of pooled naïve (unvaccinated, uninfected individuals; n>100), pooled convalescent (pooled Conv.) (n>100), convalescent and vaccinated with one dose (Conv. + 1 dose), convalescent and vaccinated with two doses (Conv. + 2 dose), vaccinated with one dose (1 dose), vaccinated with 2 doses (2 doses) of Pfizer (BNT162b2) and naïve (PCR negative confirmed). Pooled sera are samples pooled from a surveillance study of 100 individuals with (pooled convalescent) or without (pooled naïve) prior SARS-CoV-2 infection. While the rest of the samples were collected from single individuals from each category described above. (C) Binding and recognition of immobilized mammalian and plant produced RBD by conformation dependant monoclonal IgG, IgM, and IgA CR3022 antibodies and (D) their half-maximal inhibitory dilution (ID_50_) values. (E) Relative inhibition percentage of anti-SARS-CoV-2 neutralizing antibodies in blocking immobilized mammalian and plant produced RBD from binding to soluble ACE2 by snELISA. This assay is representative of technical triplicates or quadruplicates and presented as mean ± standard deviation. (F) Reciprocal ID_50_ values from (E) for mammalian- and plant-expressed RBD.

To evaluate plant-expressed RBD receptor binding kinetics, we first examined its interaction with ACE2. Fig 3 summarizes the three types of ELISA-based assays that were conducted for the functional assessment of the RBD antigens. For this purpose, binding kinetics between soluble and serially titrated ACE2 protein and immobilized plant- and mammalian-RBD were evaluated by indirect enzyme-linked immunosorbent assay (ELISA) (Fig 3A). We found that plant-expressed RBD readily binds to ACE2 with high affinity (K_D_ = 8.21 nM, R^2^ = 0.993), and at a comparable level to RBD expressed in mammalian 293F cells (K_D_ = 6.90 nM, R^2^ = 0.981) (Fig 4A). Similar binding kinetics have been reported by other studies, however the slightly lower value of the plant derived equilibrium dissociation constant (K_D_) could be attributed to minor differences in glycosylation and other post-translational modifications as a result of the different expression platforms [27–30].

Next, we examined whether plant-expressed RBD was recognized by sera from COVID-19 convalescent, partially and fully vaccinated individuals. To this effect, human sera was added to immobilized plant RBD and we probed for the binding of three classes of antibodies (IgG, IgM, and IgA) by ELISA (Figs 3B and 4B). Here, we show that plant RBD was readily detected by IgM, IgA, and IgG antibodies from naturally infected convalescent, vaccinated or convalescent and vaccinated individuals (Fig 4B). We reveal detection and high titers of IgG antibodies comparable to the mammalian-expressed RBD across all samples tested. We also report that IgM and IgA immunoreactivity to plant-expressed RBD was comparable but slightly less efficient in comparison to mammalian-derived RBD. Non-specific binding of antibodies to antigen-naïve individuals was minimal for both RBD antigens tested (Fig 4B). In contrast to previously published results [31, 32], our plant-expressed RBD was bound by conformation-dependent monoclonal IgM, IgA, and IgG CR3022 antibodies (Fig 4C). This constitutes a further indirect indication that the plant RBD antigen is folded similarly to the mammalian counterpart and exposes the conformational epitope for this antibody. While we note comparable half-maximal inhibitory dilution (ID_50_) values for IgG CR3022 (plant ID_50_ = 1.8x10^-5^, mammalian ID_50_ = 1.2x10^-5^), we did observe slightly reduced binding of the IgA CR3022 antibody to plant-derived RBD (6.9x10^-5^) compared to mammalian-expressed RBD (3.4x10^-5^), respectively (Fig 4C and 4D). Post-translational modifications such as glycosylation may also explain these small differences in IgA and IgM binding [27], although we also cannot exclude the possibility of minor unfavourable folding motifs that could contribute to these observations.

Lastly, we investigated the relative efficacy of human serum neutralizing antibodies in blocking the immobilized plant RBD’s interaction with soluble ACE2 by surrogate neutralization ELISA (snELISA) (Fig 3C) [20, 33]. As mentioned previously, most neutralizing antibodies produced by natural infection or vaccine immunity are directed to the RBD of the spike protein [3–5]. Neutralization of plant RBD-ACE2 interactions by human serum antibodies is therefore an additional indicator of adequate and functional protein folding and of neutralizing epitope accessibility on plant RBD. Serum was added to immobilized plant-expressed RBD and then binding to soluble biotinylated ACE2 receptor was evaluated (Fig 4E). We then measured the inhibition of the binding as a function of the serum dilution. Our observations indicate that human antibodies were able to bind plant-expressed RBD and neutralize its binding to the ACE2 receptor with similar kinetics to the mammalian-expressed RBD (Fig 4F). In fact, we observed generally greater ID_50_ values for the plant-expressed RBD, thereby indicating that its interactions with the ACE2 receptor were more efficiently disrupted by neutralizing antibodies.

## Discussion

We have demonstrated that plant-expressed RBD retains folding and functionality comparable to mammalian produced RBD as evaluated by CD analysis, binding to host cell receptor ACE2, recognition by the conformation-dependant monoclonal CR3022 antibody directed to RBD, and binding of polyclonal antibodies from sera of SARS-CoV-2-infected and vaccinated individuals. Although we did note a reduced ability of human IgM and IgA antibodies to bind plant-expressed RBD as compared to mammalian-expressed RBD, similar binding of IgG, which have high affinity to the target antigen, was observed. Additionally, binding of plant-expressed RBD to the ACE2 receptor was efficiently neutralized by antibodies from sera of convalescent, partially and fully vaccinated individuals. Collectively, these findings demonstrate that recombinant RBD produced in *N*. *benthamiana* exhibits suitable biochemical, structural, and antigenic features for testing as antigen in a subunit vaccine platform.

There are many advantages in developing a plant-based platform for viral antigen production, one of which is its flexibility to rapidly pivot to express antigens for emerging SARS-CoV-2 variants of concern. In six weeks, it is possible to produce the purified RBD of a new variant from an expression plasmid. The amount produced is directly linked to the number of plants grown, which can be cost-effectively scaled up to meet any laboratory requirements; growing of *N*. *benthamiana* does not require sophisticated or costly installations, making this technology broadly accessible worldwide. SARS-CoV-2 antigens are required in serological assays to measure antibodies in blood raised against the virus from exposure or induced by vaccines. Viral RBD and Spike proteins are also an integral part of ELISA-based antibody neutralization assays, as the one used in this study [20]. These types of assays are critical for measuring the potential of new variants of concern to evade vaccine immunity. Furthermore, purification yields of this RBD antigen could conceivably be doubled simply by changing the tagging system, i.e. switching out of the standard 8xhistidine tag for a FLAG-tag, a phenomenon observed in a comparative study [24]. Such a modification may also negate the cost-effectiveness of the procedure proposed in our study, however, and so further analysis is required. The expression system used to drive recombinant protein production also has a dramatic effect on purification yield, as demonstrated by the use of the potato virus X-based pEff vector [34]. Incorporating this vector into our protocol in the future could easily fast-track the scaling-up process.

As variants continue to emerge, the viral targets of vaccines and vaccination technology also need to evolve to match the pathogen’s evolutionary pace. Vaccines targeting the Spike or RBD of several variants, called multivalent vaccines, will be important to help immune responses focus on specific mutations [35, 36]. Different vaccine technologies will also have a major role to play to diversify cellular and humoral immune responses to help combat disease severity and transmission. For instance, new vaccines based on different technologies to the original mRNA and adenovirus-based vaccines are now entering and emerging from clinical trials. Protein subunit, viral vector and virus-like particle (VLP) vaccines are but a few, including a new plant-based adjuvanted VLP vaccine by Medicago [37–39]. Delivery methods such as oral and intranasal vaccine technologies that stimulate mucosal immunity specialized in reducing transmissions will also become critical for moving out of the acute stage of this pandemic [40, 41]. As customizing vaccines to new variants will be critical in keeping SARS-CoV-2 infections and transmissions in check, so will be the rapid biomanufacturing of viral antigens that are critical components for some of these vaccine technologies.

The data presented in this study therefore sets the stage for further investigating the use of cost-effective and massively scalable plant-based viral antigen in protein subunit vaccines given its comparable properties to its animal cell-expressed counterpart. Such protein antigens have already shown great promise in various research studies and clinical trials [9, 10, 42].

### Limitations of the study

The degree to which the native glycosylation profile of viral antigen is reconstituted is an important consideration to the selection of an expression system, and the expression of a heterologous (glyco)protein in a plant-based platform will yield a glycosylation profile that is distinct from that of mammalian systems [43]. Plants and animals differ in terms of both *N-* and *O*-linked glycosylation, with plants producing highly specific glycans that are absent in other eukaryotes (for example, β(1,2)-xylose and α(1,3)-fucose), and lacking others such as sialic acid [44]. Moreover, there is increasing evidence that heterologous glycoproteins may be under-glycosylated (i.e., display reduced glycan occupancy) when expressed in plants [45]. Importantly, plant-manufactured VLP-based vaccines against both influenza and SARS-CoV-2 have been demonstrated to be safe and effective in humans, despite containing plant-specific glycan structures [39, 46, 47] and ongoing initiatives to humanize the glycosylation systems of plants offer great potential [48]. In this study, it is also worth noting that the RBD of the SARS-CoV-2 Spike protein is not subject to extensive post-translational modifications and glycosylation, particularly in comparison to other heavily decorated viral proteins that are more challenging to produce in plants. Ultimately, it is yet unknown whether different glycosylation patterns are a caveat to producing our plant-derived antigen for diagnostics and subunit vaccines. Currently ongoing studies in animals will reveal the potential and efficacy of plant-expressed antigen as a subunit vaccine for SARS-CoV-2.

## Supporting information

S1 Fig (TIF)

S1 Data (XLSX)

S1 Raw images (PDF)
