# Supplementary figures and images for: Scalable agroinfiltration-based production of SARS-CoV-2 antigens for use in diagnostic assays and subunit vaccines
Source: PLoS One. 2022 Dec 14;17(12):e0277668. doi: 10.1371/journal.pone.0277668 (PMC9749978; doi:10.1371/journal.pone.0277668)

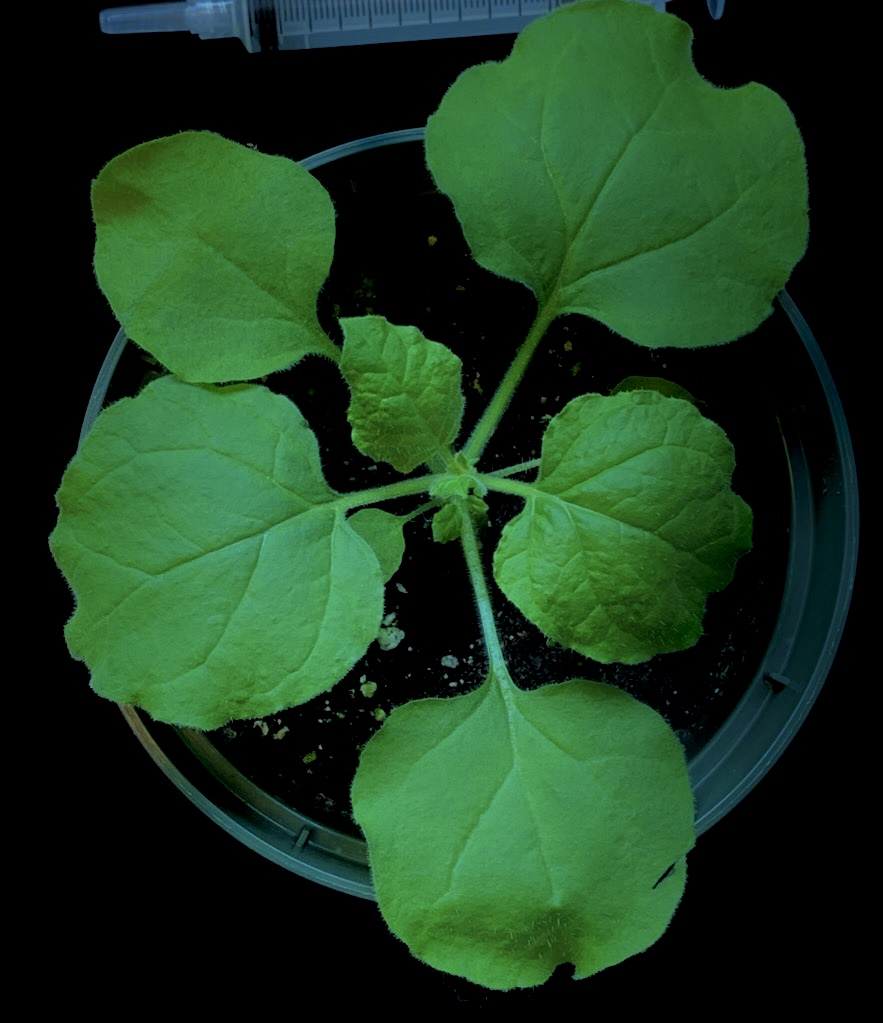

Supplement: S1 Fig — (TIF) [file pone.0277668.s001.tif]
