# Supplementary material for: Scalable agroinfiltration-based production of SARS-CoV-2 antigens for use in diagnostic assays and subunit vaccines
Source: PLoS One. 2022 Dec 14;17(12):e0277668. doi: 10.1371/journal.pone.0277668 (PMC9749978; doi:10.1371/journal.pone.0277668)
Supplement: S1 Raw images — (PDF) [file pone.0277668.s003.pdf]

Figure 1C

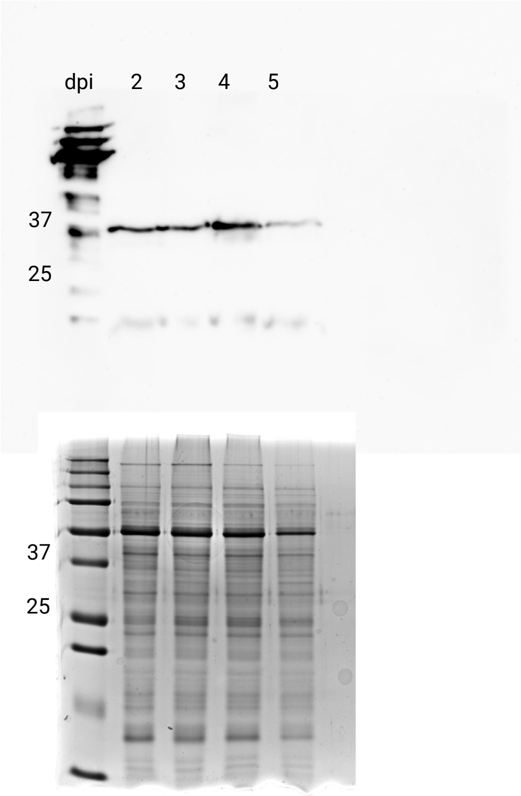

Immunoblot
Anti-his

SDS-PAGE
Coomassie Brilliant Blue staining

Figure 1D

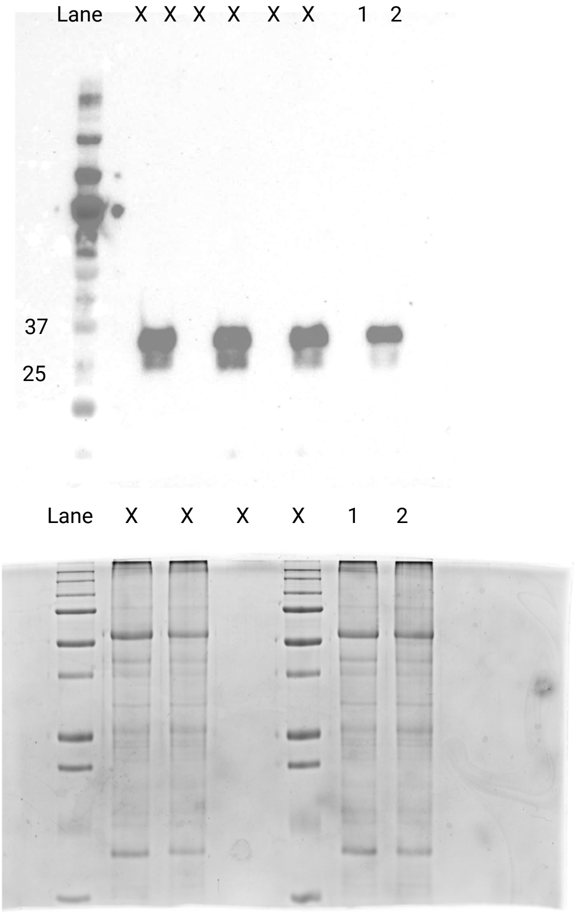

Immunoblot
Anti-his

SDS-PAGE
Coomassie Brilliant Blue staining

Figure 1E

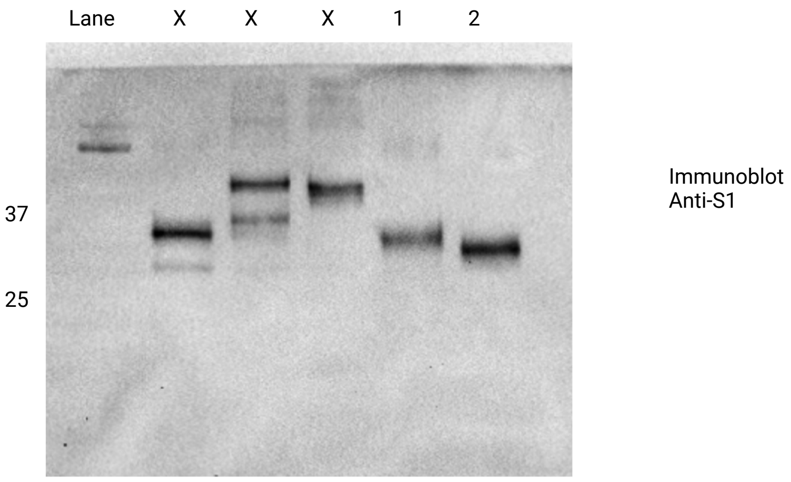

Figure 1F

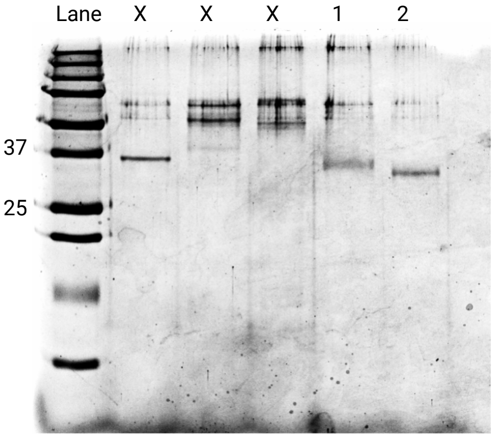

SDS-PAGE
Coomassie Brilliant Blue stained

Figure 1G

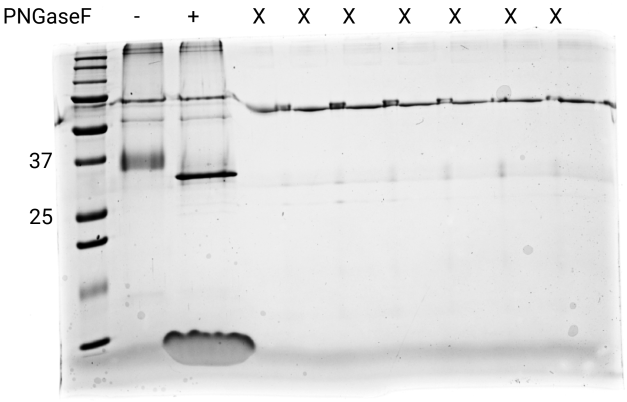

SDS-PAGE
Coomassie Brilliant Blue stained
